# Nanoscale inhomogeneity and the evolution of correlation strength in FeSe$_{1-x}$S$_x$

**DOI:** 10.1186/s40580-023-00405-2

**Published:** 2023-12-22

**Authors:** Yu Liu, Aifeng Wang, Qianheng Du, Lijun Wu, Yimei Zhu, Cedomir Petrovic

**Affiliations:** 1 Condensed Matter Physics and Materials Science Department, Brookhaven National Laboratory, Upton, NY 11973 USA; 2 Department of Materials Science and Chemical Engineering, Stony Brook University, Stony Brook, NeY 11790 USA; 3 Center for Correlated Matter and School of Physics, Zhejiang University, Hangzhou, 310058 China; 4 Present Address: College of Physics, Chongqing University, Chongqing, 401331 China; 5 Present Address: Material Science Division, Argonne National Laboratory, Lemont, IL 60439 USA

**Keywords:** Thermoelectricity, Superconductivity, Electronic correlation, Disorder

## Abstract

We report a comprehensive study of the nanoscale inhomogeneity and disorder on the thermoelectric properties of FeSe$_{1-x}$S$_x$ ($0 \le x \le 1$) single crystals and the evolution of correlation strength with S substitution. A hump-like feature in temperature-dependent thermpower is enhanced for *x* = 0.12 and 0.14 in the nematic region with increasing in orbital-selective electronic correlations, which is strongly suppressed across the nematic critical point and for higher S content. Nanoscale Se/S atom disorder in the tetrahedral surroundings of Fe atoms is confirmed by scanning transmission electron microscopy measurements, providing an insight into the nanostructural details and the evolution of correlation strength in FeSe$_{1-x}$S$_x$.

## Introduction

With a simple PbO-type structure FeSe shows a variety of complex and competing electronic phases [1], providing an ideal platform to probe the underlying physics of unconventional superconductivity (SC). Bulk FeSe is a compensated semimetal that exhibits SC with $T_\text {c}$ $\thickapprox$ 9 K [2]; the $T_\text {c}$ can be enhanced by applying physical pressure [3, 4], intercalation [5–9], ionic liquid gating [10], surface doping with alkaline metals [11, 12], isovalent S substitution in a modified hydrothermal growth [13], or in monolayer thin films on SrTiO$_3$ substrate [14–18]. Intriguingly, FeSe undergoes a tetragonal-to-orthorhombic structural (nematic) transition at $T_\text {s}$ $\approx$ 90 K without the formation of magnetic order [19–26]. This nematic phase is characterized by multiband shifts driven by orbital order that leads to Fermi-surface (FS) distortions, and it is additionally contributed by non-local Fe $d_{\text {xy}}$ orbital besides a lifting of the degeneracy of the $d_{\text {xz}}$ and $d_{\text {yz}}$ states [26–28]. Furthermore, three gaps were determined in angle-resolved specific heat measurements [29]; strong electronic correlations was proposed by detecting a Hubbard-like band in FeSe [30].

Isovalent S substitution, which maintains the nature of compensated semimetals, is an effective route for tuning electronic states and correlations in FeSe. The nematicity is strongly suppressed with S substitution, and a nematic quantum critical point (NCP) appears at $x \approx 0.17$ [31–36]. The SC gap exhibits an abrupt change across NCP [32]; meanwhile, the normal state resistivity in the vicinity of NCP shows a linear temperature dependence [37–40], signifying non-Fermi-liquid behavior due to the nematic critical fluctuations. Strange metal component can also be observed in the Hall response of FeSe$_{1-x}$S$_x$ across the NCP [41]. Recent nuclear magnetic resonance (NMR) measurements revealed that antiferromagnetic (AFM) fluctuations show a positive correlation with $T_\text {c}$ [42], although nematic fluctuations are most strongly enhanced near the QCP but without a clear correlation with the $T_\text {c}$ [34]. Raman response from fluctuations was also studied [43, 44]. Additionally, quantum oscillation experiments indicated a topological Lifshitz transition and a reduction in electronic correlations across the NCP [45].

Thermopower is an effective probe to characterize the nature and sign of transport carries as well as the correlation strength in superconductors [46–54]. In addition, correlated quantum phases may also depend on nanoscale inhomogeneity [55]. Fundamentally, thermopower is entropy per carrier. Since it is directly related to the Fermi energy, thermopower measurement will assist in understanding the band structure. In cuprates, the FS reconstruction driven by the AFM order [56] and/or charge density wave [57, 58] have been revealed by thermopower measurement.

Here we investigated the evolution of electronic correlation strength and the Se/S atomic disorder in FeSe$_{1-x}$S$_x$ by the combination of scanning transmission electron microscopy, electrical and thermoelectric transport, Hall resistivity, and specific heat measurements. Strong nanoscale Se/S disorder appears in the middle range of FeSe$_{1-x}$S$_x$, away from the NCP. Our results indicate modest electronic correlations in FeSe and their significant reduction across the NCP, confirming an orbital selective correlation [59] and its weak connection to the nanostructural details.

## Methods

High quality single crystals of Fe$_y$Se$_{1-x}$S$_x$ ($0 \le x \le 1$, $y \le 0.1$) were fabricated by the AlCl$_3$/KCl chemical vapor transport (CVT) method for $0 \le x \le 0.23$, and by the hydrothermal method for $0.4 \le x \le 1$, respectively, which are characterized as described previously [60–65] and labeled as FeSe$_{1-x}$S$_x$ ($0 \le x \le 1$) in this paper. High-resolution scanning transmission electron microscopy (STEM) imaging were carried out using the double aberration-corrected JEOL-ARM200CF microscope with high angle annular dark field (HAADF) detector and operated at 200 kV. The STEM-HAADF images were acquired with convergent semi angle of 21 mrad and collection semi angle from 67 to 275 mrad, respectively. The STEM samples were prepared by crushing the crystals and dispersed on a Lacy carbon TEM grid. The temperature-dependent in-plane resistivity $\rho$(T) and thermopower *S*(T) were measured in a quantum design PPMS-9 with standard four-probe technique. The relative error in our measurement for thermopower was below 5% based on Ni standard measured under the identical conditions. The heat capacity was measured by the heat pulse relaxation method in PPMS-9. The Hall resistivity $\rho _{\text {xy}}$(H) was measured using standard four-probe method with the current flowing in the *ab* plane and the magnetic field applied along the *c* axis. In order to effectively eliminate the longitudinal resistivity contribution due to voltage probe misalignment, the $\rho _{\text {xy}}$(H) was obtained by the difference of transverse resistance measured at positive and negative fields, i.e. $\rho _{\text {xy}} = [\rho _{\text {+H}}-\rho _{\text {-H}}]/2$. The sample dimensions were measured by an optical microscope Nikon SMZ-800 with 10 $\mu$m resolution.

## Results and Discussion

**Fig. 1:**
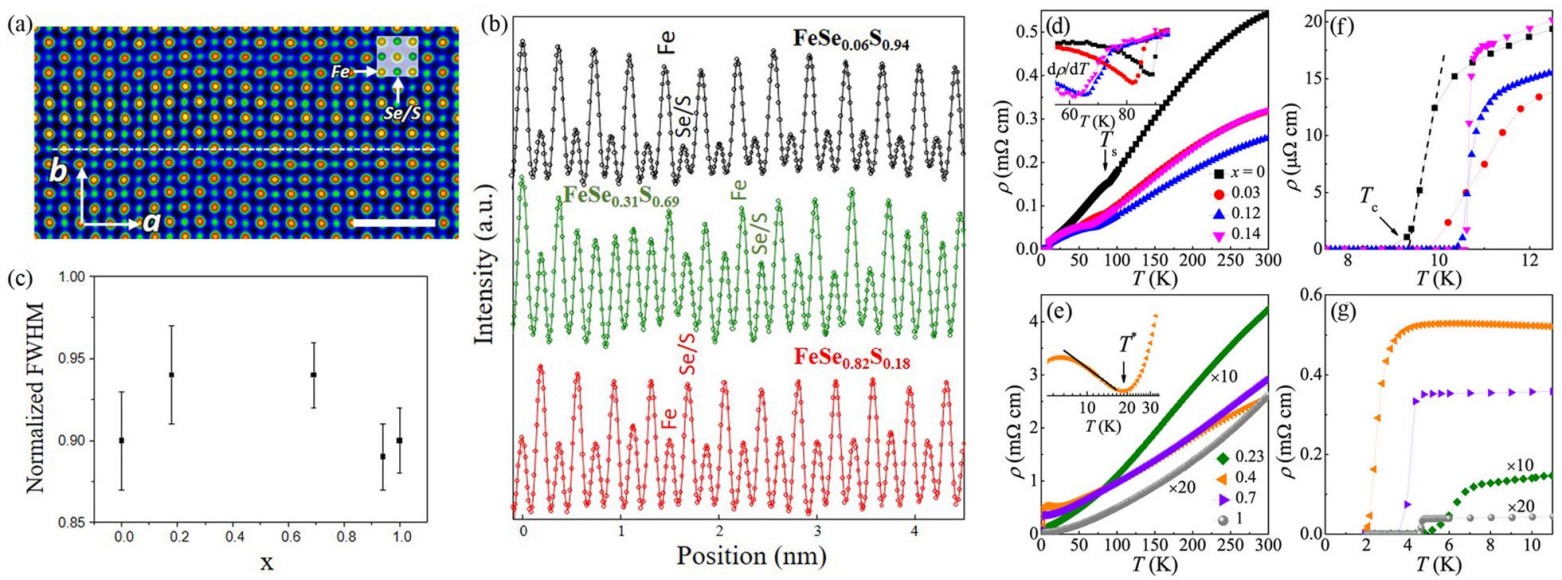
(Color online) **a** STEM-HAADF image of FeSe$_{0.06}$S$_{0.94}$ with the incident beam along the [001] direction. The [001] projection of the structure model is illustrated with yellow and green spheres representing Fe and Se/S atom, respectively. Scale bar 1nm. **b** Intensity profile (black circles in top) from the white dash scan line shown in (**a**). Two other intensity profiles from images of FeSe$_{0.31}$S$_{0.69}$ and FeSe$_{0.82}$S$_{0.18}$ are shown in green and red circles, respectively. The solid lines are the fitted curves based on Gaussian function to obtain the full width at half maximum (FWHM) of Fe and Se/S columns. **c** Normalized FWHM of Se/S columns (FWHM$_{\text {Se/S}}$/FWHM$_{\text {Fe}}$) as a function to S concentration *x* in FeSe$_{1-x}$S$_x$. **d**, **e** Temperature dependence of in-plane resistivity $\rho$(T) for FeSe$_{1-x}$S$_x$. Inset in **d** shows the $d\rho /dT$ versus *T* for the indicated samples. Inset in e shows the resistivity upturn region in log$_{10}T$ scale for *x* = 0.4. **f**, **g** The enlargement of superconducting transitions at low temperatures

High-resolution scanning transmission electron microscopy images recorded by high-angle annular dark-field detector (STEM-HAADF) indicate the presence of nanoscale inhomogeneity and strong disorder in the middle *x* range compared to FeSe and FeS [63]. The contrast of Se/S columns is weaker than that of Fe columns in high-S samples (Fig. 1a) and becomes stronger in high-Se samples [63]. From the refinement (Fig. 1b), we obtained that the full width at half-maximum (FWHM) values are 0.112(5) nm (Se/S peak) and 0.125(2) nm (Fe peak) for FeSe$_{0.06}$S$_{0.94}$, 0.106(3) nm (Se/S peak) and 0.112(2) nm (Fe peak) for FeSe$_{0.31}$S$_{0.69}$, and 0.116(4) nm (Se/S peak) and 0.124(3) nm (Fe peak) for FeSe$_{0.82}$S$_{0.18}$, respectively. We noted that the peak width of the atomic columns in STEM images depends on not only the disorder of the atomic position but also the probe size. The later may vary slightly due to the alignment of the microscope. To minimize the effect of the probe size variation, we calculate the normalized FWHM (FWHM$_{\text {Se/S}}$/FWHM$_{\text {Fe}}$). Figure 1(c) exhibits the normalized FWHM of Se/S columns, indicating that Se/S atomic positions are more disordered in the middle range of S doping at Se sites when compared to FeSe and FeS.

Figure 1(d,e) shows the temperature dependence of in-plane resistivity $\rho$(T) for FeSe$_{1-x}$S$_x$, typical metallic behavior over the entire temperature [63, 64]. The nematic transition temperature $T_s$ determined by the minima of $d\rho /dT$ is $\sim$ 89 K for FeSe; it is monotonically suppressed by S substitution [Table 1], and is absent for $x = 0.23$ and higher *x*, arising from the suppression of orbital ordering by chemical pressure [66]. The residual resistivity ratio *RRR* = $\rho$(300 K)/$\rho$(14 K) is $\sim$ 25 for FeSe, confirming low defect scattering and relatively high quality for vapor-grown FeSe crystals [67], which can be further optimized to $> 40$ [68, 69]. As *x* is increased from 0, the value of RRR first decreases to 14.6 and 15.5 for *x* = 0.12 and *x* = 0.14, respectively, and then increases to 25.5 for $x = 0.23$ when $T_s$ is completely suppressed (Fig. 1e). With increasing *x*, FeSe$_{1-x}$S$_x$ with *x* = 0.4 and 0.7 show rather small values of *RRR* $\sim$ 5 and 8, respectively, due to the enhancement of anion height disorder-related scattering [63]. Then the *RRR* dramatically increases to $\sim 53.2$ for pure FeS, higher than the reported 10 $\sim$ 46 for hydrothermal-synthesized FeS crystals [70–72]. Crystals obtained by hydrothermal technique usually contain more impurities, however crystals near *x* = 0.5 and *x* = 1 crystal are created using the same hydrothermal crystal growth. Whereas *x* = 0.4 and *x* = 0.7 have low RRR, *x* = 1 has much higher RRR. Indeed, it was observed that FeS is a very good metal with low defect scattering and it shows quantum oscillations just like FeSe crystals made by cvt method [73, 74]. This suggests that the differences observed as *x* is varied between 0 and 1 are unrelated to synthesis process-related disorder. We note that an anomalous resistivity upturn with a characteristic temperature $T^*$ emerges for $0.3< x < 0.7$ as observed in our previous work [63, 64], similar in FeSe$_{1-x}$S$_x$ thin films [75]. Below $T^*\sim 18$ K for a representative sample *x* = 0.4 (inset in Fig. 1e), the resistivity obeys a log$_{10}T$-dependence before $T_\text {c}$, which might be related to spin fluctuations caused by strong nanoscale crystallographic disorder [35]. Figure 1(f,g) shows the $\rho$(T) curves at low temperatures for FeSe$_{1-x}$S$_x$; an abrupt resistivity drop can be clearly seen, signaling the onset of SC. Zero resistivity is observed at $T_\text {c}$ = 9.3(1) K for FeSe; the $T_c$ shows a maximum value of 10.6(1) K for $x = 0.12$ within the nematic region, a minimum value of 2.1(1) K for *x* = 0.4, and increases again to $\sim$ 4.5(1) K for FeS [Table 1]. This two-dome-like superconducting phase diagram of FeSe$_{1-x}$S$_x$ was plotted in our previous work [63, 64], and similar feature was also observed in several other unconventional superconductors [76–79].

**Fig. 2:**
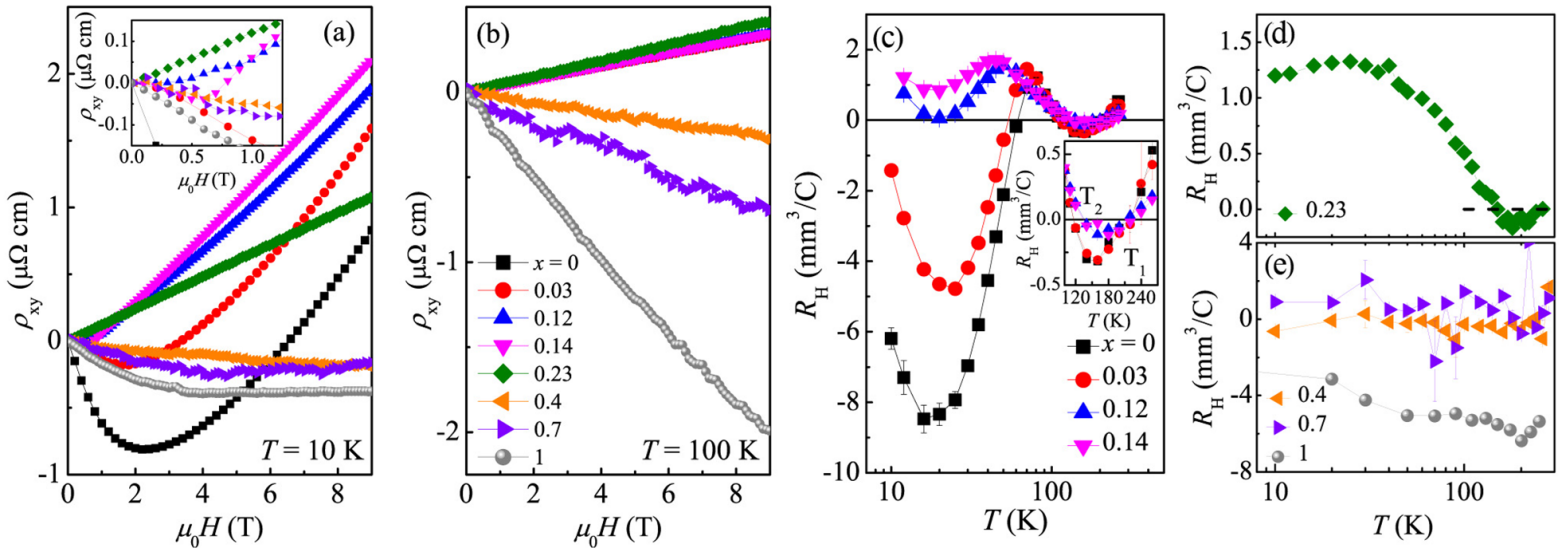
(Color online) Field dependence of Hall resistivity $\rho _{\text {xy}}$ at representative temperatures **a** $T =$ 10 K and **b** $T =$ 100 K for FeSe$_{1-x}$S$_x$. Inset in a shows the low-field data for the indicated samples. **c**–**e** Temperature dependence of low-field Hall coefficient $R_\text {H}$ for FeSe$_{1-x}$S$_x$

Figure 2a presents the magnetic field dependence of Hall resistivity $\rho _{\text {xy}}$ at *T* = 10 K for FeSe$_{1-x}$S$_x$. A strong nonlinear feature is observed for FeSe, and it is gradually weakened with increasing *x* in high-Se region. Linear field dependence of $\rho _{\text {xy}}$ is seen for $x =$ 0.4 and 0.7, and then it evolves into nonlinear again for high-S samples, confirming multi-band and delicate tuning in carriers. The Hall effect is dominated by hole carriers at high fields with a change of sign at low fields for $x \le 0.12$; the sign change is absent for higher *x* samples (inset in Fig. 2a). We should note that the low-field negative Hall coefficients in CVT-grown FeSe, arising from a small minority of highly mobile electron carriers [80–82], was not observed in hydrothermal FeSe [36], due to more disorder induced by hydrothermal method. With increasing *x*, the Hall effect is dominated by electron carriers for high-S hydrothermal samples. Figure 2(b) shows the $\rho _{\text {xy}}$ curves at $T = 100$ K with linear-field dependence for all samples; the slope is positive with slight change for $0 \le x \le 0.23$, confirming isovalent S substitution, which changes into negative with dominant electrons for high-S hydrothermal samples. Figure 2(c-e) displays the temperature-dependent Hall coefficient $R_\text {H}$ with error bars determined from linear fits of $\rho _{\text {xy}}$ below 1 T for FeSe$_{1-x}$S$_x$. Within the nematic region $0 \le x < 0.17$ (Fig. 2c), the $R_\text {H}$ shows a W-shape in *T*-dependence and changes in sign several times, reflecting multi-band nature. The values of $R_\text {H}$ are small at high temperatures, which can be understood by a compensated multi-band model. The sign change of $R_\text {H}$ above 100 K (Fig. 2c inset, $T_1$ and $T_2$) suggests that at some temperatures the holes are more mobile and at other temperatures the electrons are. With decreasing temperature, a humplike feature was observed with its peak temperature gradually suppressed by S substitution, corresponding to the suppressed nematic transition. The $R_\text {H}$ decreases below the peak temperature due to an enhanced contribution of electron-type carriers which becomes dominant for *x* = 0 and 0.03 while it stays positive for *x* = 0.12 and 0.14; then it increases again below 20 K. Across the NCP, the behavior is clearly different for higher *x*. For *x* = 0.23 (Fig. 2d), the $R_\text {H}$ also changes in sign twice at high temperatures but it gradually increases and saturates at $\sim$ 1.25 mm$^3$/C below 30 K. The values of $R_\text {H}$ are of the same order or smaller for *x* = 0.4 and 0.7 (Fig. 2e) with weak temperature dependence when compared to $x = 0.23$, however, it is not clear about the sign change due to larger error bars. This roughly points to larger values of carrier density in hydrothermal-grown *x* = 0.4 and 0.7. For FeS, the $R_\text {H}$ is negative in the whole temperatures range, in agreement with previous report [72].

**Fig. 3:**
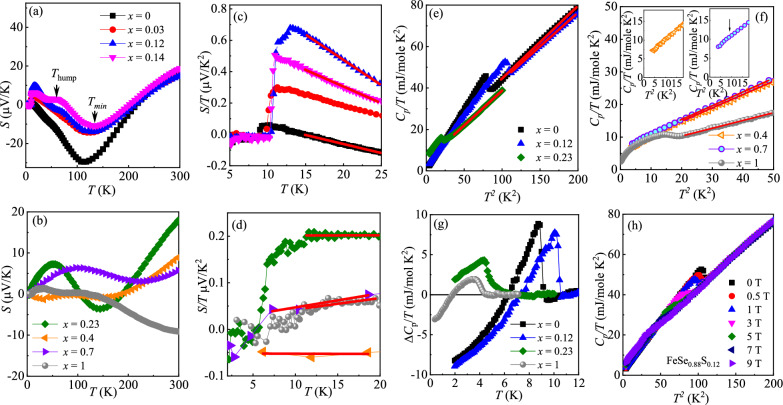
(Color online) **a**, **b** Temperature dependence of in-plane thermopower *S*(T) for FeSe$_{1-x}$S$_x$. Inset in a shows the *dS*/*dT* versus *T* curves with arrows defining $T_{\text {hump}}$ and $T_{\text {min}}$, respectively. **c**, **d** The *S*/*T* versus *T* curves at low temperatures with linear fits before $T_\text {c}$. **e**, **f** Temperature dependence of specific heat $C_\text {p}/T$ vs $T^2$ with linear fits above $T_\text {c}$ for FeSe$_{1-x}$S$_x$. **g** The $\Delta C_\text {p}/T$ versus *T* subtracted by the linear fit for the indicated samples. **h** The $C_\text {p}/T$ versus $T^2$ at various fields for FeSe$_{0.88}$S$_{0.12}$. Inset in (h) shows the field induced electronic specific heat $\Delta \gamma _\text {e}$ versus $\mu _0 H$ with a fit to the Volovik relation $\Delta \gamma _\text {e} = A\sqrt{H}$

Figure 3(a,b) shows the temperature dependence of in-plane thermopower. For FeSe, the *S*(T) is positive $\sim$ 18 $\mu$V/K at 300 K, changes sign at 215 K, goes through a broad negative minimum $\sim$ -30 $\mu$V/K at 110 K, and changes sign again below $\sim$ 15 K, similar to the reported features in polycrystalline FeSe [83–85]. This is indicative of a multiband system with competition of almost compensated hole and electron bands. At high temperatures, the positive values of *S*(T) dominated by hole band are almost unchanged with *x* within the nematic region (Fig. 3a), confirming that the effect of S substitution is in chemical pressure rather than in charge carrier doping. With decreasing temperature, the *S*(T) changes sign at 190 $\sim$ 215 K for *x* = 0 - 0.14, corresponding to the first sign change of $R_\text {H}$ at $T_1$ (Fig. 2c inset); the $T_{\text {min}}$ for minima thermpower (defined by *dS*/*dT* = 0 in Fig. 3a inset) shifts from 110 to 145 K with increasing *x*, agreeing well with the second sign change at $T_2$ in $R_\text {H}$ (Fig. 2c inset) [80, 81].

Interestingly, a weak hump-like feature occurs around $T_{\text {hump}} \sim$ 45(5) K defined by the local maximum in *dS*/*dT* for FeSe (Fig. 4a inset); it is rather weak for *x* = 0.03 and becomes more apparent at $T_{\text {hump}} \sim$ 64(2) K and 51(1) K for *x* = 0.12 and 0.14, respectively, when RRR is decreased. The $T_{\text {hump}}$ is close to the $T_\text {s}$ for *x* = 0.12 and 0.14, but if it is due to the nematic transition it should be more apparent in undoped FeSe at $T_\text {s} \sim 89$ K, which is not seen here. If we consider the local minimum in *dS*/*dT* as the onset temperature of the hump, it is enhanced from 74(3) K for $x = 0$ to 88(11) K for $x = 0.14$, in contrast to the suppression of nematic transition with increasing S substitution. Additionally, it would not be pure carrier diffusion effect that would also produce more apparent feature for *x* = 0 and 0.03 due to pronounced variation in $R_\text {H}$ when compared to *x* = 0.12 and 0.14 (Fig. 2c). In the temperature-dependent angle-resolved photoemission spectroscopy (ARPES) measurements of detwinned FeSe, the disappearance of a band can be inferred at this particular energy at $T \sim$ 60 K [86], i.e. a Lifshitz transition as a function of temperature in FeSe [28]. However, we can not exclude the phonon drag effect although there is no peak at $\Theta _\text {D}/5 \sim$ 44.6(2) K with the Debye temperature $\Theta _\text {D} =$ 223(1) K for FeSe (see below). In addition, DFT and DMFT calculations of pressurized FeSe predict two topological transitions [87]. With S substitution, there is strong increase in the interlayer warping in FeSe$_{1-x}$S$_x$, as the conducting layers come closer together due to chemical pressure, yet we also note the presence of disorder as evidenced in decreased RRR. The high-resolution ARPES detected a small inner hole pocket for *x* = 0.12, which is pushed below the Fermi level in pure FeSe [66]. The Shubnikov-de Haas oscillation experiment shows a sudden change in extremal areas of Fermi surface pockets near $x \sim 0.15$ [45], i.e. a S-substitution-induced Lifshitz transition that is generated by a sizeable non-local Fe $d_{\text {xy}}$ nematic term at the corner of the Brillouin zone [28]. The S-substitution-induced Lifshitz transition with $x \sim$ 0.12 - 0.15 explains a more visible hump feature in the *S*(T) when compared to FeSe. Therefore, we propose the S-substitution-induced Lifshitz transition possibly plays a role for the pronounced hump in *S*(T) for *x* = 0.12 and 0.14, perhaps combined with the phonon drag effect. Moreover Lifshitz transition associated with sharp changes in the Fermi surface should be facilitated with the nematic order suppression, in line with our observatons [28], whereas disorder effects from sulfur substitution (Fig. 1a–c) should decrease phonon drag contribution [88]. We should note that the ARPES detecteition between 180 and 282 K for FeSe$_{0.945}$S$_{0.055}$ [89] is not reflected in our thermopower measurement for *x* = 0.03. Outside the nematic region, the value of *S*(300 K) decreases with increasing *x* and finally becomes a negative value of $\sim$ -9 $\mu$V/K for FeS Fig. 3b, in line with its negative value of $R_\text {H}$.

Figure 3c, d shows the temperature-dependent *S*/*T* at low temperatures. Linear *T*-dependence observed above $T_\text {c}$ sharply decreases to zero below $T_\text {c}$, and the values of $T_\text {c}$ agrees with the resistivity results. Zero-temperature extrapolations of *S*/*T* are summarized in Table 1. The thermopower *S* is usually given by [90–94],

1 $$\begin{aligned} \frac{S}{T} = \pm \frac{\pi ^2}{2}\frac{k_\text {B}}{e}\frac{1}{T_\text {F}} = \pm \frac{\pi ^2}{3}\frac{k_\text {B}^2}{e}\frac{N(\varepsilon _\text {F})}{n}, \end{aligned}$$

where *e* is the electron charge, $k_\text {B}$ is the Boltzmann constant, $T_\text {F}$ is the Fermi temperature related to the Fermi energy $\varepsilon _\text {F}$ and density of states $N(\varepsilon _\text {F})$ as $N(\varepsilon _\text {F})$ = $3n/2\varepsilon _\text {F}$ = $3n/2k_\text {B}T_\text {F}$, and *n* is the carrier concentration. In a multiband system, it gives the upper limit of $T_\text {F}$ of dominant band. Therefore we can extract $T_\text {F}$ = $2.52(5)\times 10^3$ K for FeSe, decreases to $0.38(1)\times 10^3$ K for $x = 0.12$ and then increases to $2.03(6)\times 10^3$ K for $x = 0.23$ [Table 1]. The ratio of $T_\text {c}/T_\text {F}$ typically characterizes the correlation strength in superconductors. For example, the $T_\text {c}/T_\text {F}$ is close to 0.1 in Fe$_{1+y}$Te$_{1-x}$Se$_x$ [49], pointing to the importance of electronic correlation, while it is $\sim$ 0.02 in a BCS superconductor LuNi$_2$B$_2$C [49]. The value of $T_\text {c}/T_\text {F}$ is $\sim$ 0.0276(5) for FeSe$_{0.88}$S$_{0.12}$, indicating modest correlations, comparable to K$_x$Fe$_{2-y}$Se$_2$ $\sim$ 0.04 [52]. Moreover, the derived values of $T_\text {c}/T_\text {F}$ [Table 1] show a significant reduction in electronic correlations across the NCP for higher *x* samples.

Figure 3(e,f) shows the heat capacity $C_\text {p}/T$ as a function of $T^2$ in zero field. A clear jump is observed at $T_\text {c}$, in agreement with the transport measurements, indicating bulk SC except for *x* = 0.4 where resistivity reaches zero just below 2 K (Fig. 1e). The data just above $T_\text {c}$ can be well fitted by $C_\text {p}/T = \gamma + \beta T^2$, where the first term is the Sommerfeld electronic specific heat coefficient and the second term is low-temperature limit of lattice heat capacity. The derived $\gamma$ as well as the Debye temperature $\Theta _\text {D} = (12\pi ^4NR/5\beta )^{1/3}$, where *N* = 2 is the number of atoms per formula unit, and *R* = 8.314 J mol$^{-1}$ K$^{-1}$ is the molar gas constant, are summarized in Table 1. According to the McMillan formula for electron–phonon mediated SC, the electron–phonon coupling constant $\lambda$ can be deduced by

2 $$\begin{aligned} T_c=\frac{\Theta _D}{1.45}\exp \left[-\frac{1.04(1+\lambda )}{\lambda -\mu ^{*}(1+0.62\lambda )}\right], \end{aligned}$$

where $\mu ^{*}\approx$ 0.13 is the common value for Coulomb pseudo-potential [95]. By using the $T_c$ = 9.3(1) K and $\Theta _D$ = 223(1) K for FeSe, we obtain $\lambda \approx$ 0.91(2), a typical value of an intermediate-coupled BCS superconductor. However, the specific heat jump at $T_c$ subtracted by the liner fit (Fig. 3g), $\Delta C_p$/$\gamma T_c\approx$ 0.92(3), is somehow smaller than the weak coupling value of 1.43 [95].

**Table 1:** A set of parameters derived from in-plane resistivity $\rho$(T), thermopower *S*(T), and specific heat $C_\text {p}$(T) with errors from fitting or calculation for FeSe$_{1-x}$S$_x$

*x*	$T_\text {s}$	$T_\text {c}$	*S*/*T*	$T_\text {F}$	$T_\text {c}/T_\text {F}$	$\gamma$	$\Theta _\text {D}$	$\lambda$	*q*	$m^*$	$k_\text {F}$	$\nu _\text {F}$
	(K)	(K)	($\mu$V/K$^2$)	($\times 10^3$ K)	($\times 10^{-3}$)	(mJ/mol K$^2$)	(K)			($\times m_e$)	(1/nm)	(km/s)
0	89(1)	9.3(1)	0.169(3)	2.52(5)	3.69(3)	9.5(3)	223(1)	0.91(2)	1.72(2)	6.4(1)	6.03(3)	109(1)
0.03	82(1)	9.9(1)	0.459(4)	0.93(1)	10.6(1)							
0.12	65(1)	10.6(1)	1.118(5)	0.38(1)	27.6(5)	11.0(2)	228(1)	0.97(2)	9.8(1)	13.4(1)	3.39(2)	29(1)
0.14	61(2)	10.5(1)	0.730(6)	0.58(1)	18.3(1)							
0.23		5.2(1)	0.201(6)	2.12(6)	2.45(2)	5.2(1)	226(1)	0.71(2)	3.73(4)	4.6(1)	4.70(2)	118(2)
0.4		2.1(1)	-0.05(1)	8.5(17)	0.25(4)	6.7(2)	212(1)	0.56(2)	0.7(1)	3.5(1)	8.2(1)	202(4)
0.7		3.7(1)	0.017(8)	25(12)	0.15(7)	7.0(1)	210(1)	0.65(2)	0.2(1)	2.5(1)	12(2)	556(70)
1		4.5(1)	0.018(7)	24(10)	0.19(7)	5.5(1)	254(1)	0.65(2)	0.3(1)	2.2(1)	11(1)	579(26)

The electronic specific heat can be also expressed as:

3 $$\begin{aligned} \gamma = \frac{\pi ^2}{2}k_\text {B}\frac{n}{T_\text {F}} = \frac{\pi ^2}{3}k_\text {B}^2N(\varepsilon _\text {F}). \end{aligned}$$

Combining equations (1) and (3) yields: $S/T = \pm \gamma /ne$, where the units are V K$^{-1}$ for *S*, J K$^{-2}$ m$^{-3}$ for $\gamma$, and m$^{-3}$ for *n*, respectively. This relation was shown to hold in the *T* = 0 limit for a large variety of materials, even in the presence of strong correlations, including heavy fermion metals and cuprates [92]. Then we can define a dimensionless quantity

4 $$\begin{aligned} q=\frac{S}{T}\frac{N_\text {A}e}{\gamma }, \end{aligned}$$

where $N_\text {A}e = 96440$ C/mol is the Faraday constant. The *q* gives the number of carriers per formula unit [92]. The derived *q* is $\sim$ 1.72(2) for FeSe, which increases to 9.8(1) for $x = 0.12$ and then decreases to 3.73(4) for $x = 0.23$; the smaller value of *q* for higher *x* samples indicates larger numbers of carriers [Table 1]. Given the volume of unit cell $\sim$ 0.078487 nm$^3$ for FeSe, we obtain the carrier density per volume $n \approx 7.4(1) \times 10^{21}$ cm$^{-3}$ and the Fermi momentum $k_\text {F} = (3\pi ^2n)^{1/3} \approx 6.03(3)$ nm$^{-1}$ for FeSe; changing to $n \approx 1.32(2) \times 10^{21}$ cm$^{-3}$ and $k_\text {F} \approx 3.39(2)$ nm$^{-1}$ for $x = 0.12$ and $n \approx 3.51(4) \times 10^{21}$ cm$^{-3}$ and $k_\text {F} \approx 4.70(2)$ nm$^{-1}$ for $x = 0.23$, respectively. For samples with higher *x*, the carrier concentration $n \approx 1.85(4) \sim 6(3) \times 10^{22}$ cm$^{-3}$ and $k_\text {F} \approx 8.2(1) \sim 12(2)$ nm$^{-1}$ also increase.

The effective mass $m^*$ derived from $k_\text {B}T_\text {F} = \hbar ^2 k_\text {F}^2/2\,m^*$, evolves from 6.4(1) $m_e$ for FeSe to 13.4(1) $m_e$ for $x = 0.12$, then monotonically decrease to 2.2(1) $m_e$ for FeS, consistent with the evolution of correlation strengths with S substitution. The Fermi velocity $\nu _\text {F}$ obtained by using $\hbar k_\text {F} = m^*\nu _\text {F}$; it decreases from 109(1) km s$^{-1}$ for FeSe to 29(1) km s$^{-1}$ for $x = 0.12$, and then monotonically increases to 579(26) km s$^{-1}$ for FeS. The quasiparticle mass is larger inside the nematic phase, indicative of a strongly correlated state; it becomes suppressed outside the nematic region. The experimentally observed changes in the FS topology, together with the varying degree of electronic correlations, will change the balance of electronic interactions in multiband FeSe$_{1-x}$S$_x$. Moreover, similar nanoscale disorder is observed near NCP (*x* = 0.18) and outside it (*x* = 0.69) (Fig. 1c). This suggests that nematic correlations in critical region are only weakly perturbed by atomic bond distance disorder. Furthermore, this may also imply that enhanced scattering associated with Mooij law violation is associated with magnetic rather than nematic fluctuations away from NCP [64].

Figure 3(h) shows the specific heat under various magnetic fields for FeSe$_{0.88}$S$_{0.12}$ with the highest $T_\text {c} = 10.6(1)$ K [63, 64], where the superconducting anomaly gradually shifts to lower temperatures with lower amplitude. It is clear that normal state specific heat does not depend on the field. Then we derive the field dependence of $\Delta \gamma$(H) = [$C_\text {p}$(H)-$C_\text {p}$(0)]/*T* by extending the low-temperature specific heat to 0 K at different magnetic fields as plotted in Fig. 4d inset. The $\Delta \gamma$(H) is close to the Volovik relation, namely $\Delta \gamma$(H) $\propto$ $\sqrt{H}$ [96], indicating the presence of gap nodes in FeSe$_{0.88}$S$_{0.12}$. However, we can not distinguish whether the gap nodes are induced by the sign change of gap as in a *d*-wave case, or by the accidental nodes as theoretically predicted for FeAs- and FeSe-based superconductors [97]. This calls for further investigation of heavily S-substituted FeSe.

## Conclusions

In summary, we have comprehensively investigated the transport and thermodynamic properties of FeSe$_{1-x}$S$_x$ superconductors. The hump feature in thermopower is more pronounced in S-substituted samples *x* = 0.12 and 0.14, revealing an interplay of the increased chemical pressure, disorder and the orbital selective electronic correlations that are strongly suppressed in FeSe$_{1-x}$S$_x$ across the NCP and are not driven by the nanoscale Se/S atom disorder.

## Data Availability

The data that support the findings of this study are available from the corresponding author upon reasonable request.

## References

[1] Coldea AI, Watson MD (2018). The key ingredients of the electronic structure of FeSe. Annu. Rev. Condens. Matter Phys..

[2] Hsu F, Luo J, Yeh K, Chen T, Huang T, Wu PM, Lee Y, Huang Y, Chu Y, Yan D, Wu M (2008). Superconductivity in the PbO-type structure $$\alpha$$-FeSe. Proc. Natl. Acad. Sci. U.S.A..

[3] Medvedev S, McQueen TM, Troyan IA, Palasyuk T, Eremets MI, Cava RJ, Naghavi S, Casper F, Ksenofontov V, Wortmann G, Felser C (2009). Electronic and magnetic phase diagram of $$\beta$$-Fe$$_{1.01}$$Se with superconductivity at 3.67 K under pressure. Nat. Mater..

[4] Terashima T, Kikugawa N, Kasahara S, Watashige T, Shibauchi T, Matasuda Y, Wolf T, Böhmer AE, Hardy F, Meingast C, Löhneysen HV, Uji S (2015). Pressure-induced antiferromagnetic transition and phase diagram in FeSe. J. Phys. Soc. Jpn..

[5] Guo J, Jin S, Wang G, Wang S, Zhu K, Zhou T, He M, Chen X (2010). Superconductivity in the iron selenide K$$_x$$Fe$$_2$$Se$$_2$$ ($$0 \le x \le 1.0$$). Phys. Rev. B.

[6] Dagotto E (2013). Colloquium: the unexpected properties of alkali metal iron selenide superconductors. Rev. Mod. Phys..

[7] Burrard-Lucas M, Free DG, Sedlmaier SJ, Wright JD, Cassidy SJ, Hara Y, Corkett AJ, Lancaster T, Baker PJ, Blundell SJ, Clarke SJ (2013). Enhancement of superconducting transition temperature of FeSe by intercalation of a molecular spacer layer. Nat. Mater..

[8] Lu XF, Wang NZ, Wu H, Wu YP, Zhao D, Zeng XZ, Luo XG, Wu T, Bao W, Zhang GH, Huang FQ, Huang QZ, Chen XH (2015). Coexistence of superconductivity and antiferromagnetism in (Li$$_{0.8}$$Fe$$_{0.2}$$)OHFeSe. Nat. Mater..

[9] Dong X, Zhou H, Yang H, Yuan J, Jin K, Zhou F, Yuan D, Wei L, Li J, Wang X, Zhang G, Zhao Z (2015). Phase diagram of (Li$$_{1-x}$$Fe$$_x$$)OHFeSe: a bridge between iron selenide and arsenide superconductors. J. Am. Chem. Soc..

[10] Lei B, Cui JH, Xiang ZJ, Shang C, Wang NZ, Ye GJ, Luo XG, Wu T, Sun Z, Chen XH (2016). Evolution of high-temperature superconductivity from a low-$$T_c$$ phase tuned by carrier concentration in FeSe thin flakes. Phys. Rev. Lett..

[11] Wen CH, Xu HC, Chen C, Huang ZC, Lou X, Pu YJ, Song Q, Xie BP, Abdel-Hafiez M, Chareev DA, Vasiliev AN, Peng R, Feng DL (2016). Anomalous correlation effects and unique phase diagram of electron-doped FeSe revealed by photoemission spectroscopy. Nat. Commun..

[12] Seo JJ, Kim BY, Kim BS, Jeong JK, Ok JM, Kim JS, Denlinger JD, Mo SK, Kim C, Kim YK (2016). Superconductivity below 20 K in heavily electron-doped surface layer of FeSe bulk crystal. Nat. Commum..

[13] Sun R, Jin S, Deng J, Hao M, Zhong X, Ma Y, Li M, Chen X (2021). Chemical pressure boost record-high superconductivity in van der Waals materials FeSe$$_{1-x}$$S$$_x$$. Adv. Funct. Mater..

[14] Wang Q-Y, Li Z, Zhang W-H, Zhang Z-C, Zhang J-S, Li W, Ding H, Ou Y-B, Deng P, Chang K, Wen J, Song C-L, He K, Jia J-F, Ji S-H, Wang Y-Y, Wang L-L, Chen X, Ma X-C, Xue Q-K (2012). Interface-induced high-temperature superconductivity in single unit-cell FeSe films on SrTiO$$_3$$. Chin. Phys. Lett..

[15] Tan S, Zhang Y, Xia M, Ye Z, Chen F, Xie X, Peng R, Xu D, Fan Q, Xu H, Jiang J, Zhang T, Lai X, Xiang T, Hu J, Xie B, Feng D (2013). Interface-induced superconductivity and strain-dependent spin density waves in FeSe/SrTiO$$_3$$ thin films. Nat. Mater..

[16] He S, He J, Zhang W, Zhao L, Liu D, Liu X, Mou D, Ou Y-B, Wang Q-Y, Li Z, Wang L, Peng Y, Liu Y, Chen C, Yu L, Liu G, Dong X, Zhang J, Chen C, Xu Z, Chen X, Ma X, Xue Q, Zhou XJ (2013). Phase diagram and electronic indication of high-temperature superconductivity at 65 K in single-layer FeSe films. Nat. Mater..

[17] Lee JJ, Schmitt FT, Moore RG, Johnston S, Cui Y-T, Li W, Yi M, Liu ZK, Hashimoto M, Zhang Y, Lu DH, Devereaux TP, Lee D-H, Shen Z-X (2014). Interfacial mode coupling as the origin of the enhancement of $$T_c$$ in FeSe films on SrTiO$$_3$$. Nature.

[18] Ge JF, Liu ZL, Liu C, Gao CL, Qian D, Xue QK, Liu Y, Jia JF (2015). Superconductivity above 100 K in single-layer FeSe films on doped SrTiO$$_3$$. Nat. Mater..

[19] Shibauchi T, Hanaguri T, Matsuda Y (2020). Exotic superconducting states in FeSe-based materials. J. Phys. Soc. Jpn..

[20] McQueen TM, Williams AJ, Stephens PW, Tao J, Zhu Y, Ksenofontov V, Casper F, Felser C, Cava RJ (2009). Tetragonal-to-orthorhombic structural phase transition at 90 K in the superconductor Fe$$_{1.01}$$Se. Phys. Rev. Lett..

[21] S.-H. Baek, D. V. Efremov, J. M. Ok, J. S. Kim, Jeroen van den Brink, B. Büchner, Orbital-driven nematicity in FeSe, Nat. Mater. **14**, 210 (2015) 10.1038/nmat4138 25384167

[22] Böhmer AE, Arai T, Hardy F, Hattori T, Iye T, Wolf T, Löhneysen HV, Ishida K, Meingast C (2015). Origin of the tetragonal-to-orthorhombic phase transition in FeSe: a combined thermodynamic and NMR study of nematicity. Phys. Rev. Lett..

[23] Mukherjee S, Kreisel A, Hirschfeld PJ, Anderson BM (2015). Model of electronic structure and superconductivity in orbitally ordered FeSe. Phys. Rev. Lett..

[24] Chubukov AV, Fernandes RM, Schmalian JJ (2015). Origin of nematic order in FeSe. Phys. Rev. B.

[25] Glasbrenner JK, Mazin II, Jeschke HO, Hirschfeld PJ, Fernandes RM, Valenti R (2015). Effect of magnetic frustration on nematicity and superconductivity in iron chalcogenides. Nat. Phys..

[26] Coldea AI (2021). Electronic nematic states tuned by isoelectronic substitution in bulk FeSe$$_{1-x}$$S$$_x$$. Front. Phys..

[27] Watson MD, Kim TK, Haghighirad AA, Davies NR, McCollam A, Narayanan A, Blake SF, Chen YL, Ghannadzadeh S, Schofield AJ, Hoesch M, Meingast C, Wolf T, Coldea AI (2015). Emergence of the nematic electronic state in FeSe. Phys. Rev. B.

[28] Rhodes LC, Böker J, Müller MA, Eschrig M, Eremin IM (2021). Non-local $$d_{xy}$$ nematicity and the missing electron pocket in FeSe. NPJ Quantum Mater..

[29] Sun Y, Kittaka S, Nakamura S, Sakakibara T, Irie K, Nomoto T, Machida K, Chen J, Tamegai T (2017). Gap structure of FeSe determined by angle-resolved specific heat measurements in applied rotating magnetic field. Phys. Rev. B.

[30] Watson MD, Blake SF, Haghighirad AA, Hoesch M, Kim TK, Coldea AI, Valentí R (2017). Formation of Hubbard-like bands as a fingerprint of strong electron-electron interactions in FeSe. Phys. Rev. B.

[31] Hafiez MA, Zhang YY, Cao ZY, Duan CC, Karapetrov G, Pudalov VM, Vlasenko VA, Sadakov AV, Knyazev DA, Romanova TA, Chareev DA, Volkova OS, Vasiliev AN, Chen XJ (2015). Superconducting properties of sulfur-doped iron selenide. Phys. Rev. B.

[32] Hosoi S, Matsuura K, Ishida K, Wang H, Mizukami Y, Watashige T, Kasahara S, Matsuda Y, Shibauchi T (2016). Nematic quantum critical point without magnetism in FeSe$$_{1-x}$$S$$_x$$ superconductors. Proc. Natl. Acad. Sci. U.S.A..

[33] Reiss P, Watson MD, Kim TK, Haghighirad AA, Woodruff DN, Bruma M, Clarke SJ, Coldea AI (2017). Suppression of electronic correlations by chemical pressure from FeSe to FeS. Phys. Rev. B.

[34] Wiecki P, Rana K, Böhmer AE, Lee Y, Bud’ko SL, Canfield PC, Furukawa Y (2018). Persistent correlation between superconductivity and antiferromagnetic fluctuations near a nematic quantum critical point in FeSe$$_{1-x}$$S$$_x$$. Phys. Rev. B.

[35] Yi X, Xing X, Qin L, Feng J, Li M, Zhang Y, Meng Y, Zhou N, Sun Y, Shi ZX (2021). Hydrothermal synthesis and complete phase diagrams of FeSe$$_{1-x}$$S$$_x$$ ($$0 \le x \le 1$$) single crystals. Phys. Rev. B.

[36] Liu WH, Yi XL, Li WC, Xing XZ, Zhao HJ, Xu MX, Shi ZX (2022). Transport properties and phase diagrams of FeSe$$_{1-x}$$S$$_x$$ ($$0 \le x \le 1$$) single crystals. J. Alloys Compd..

[37] Lederera S, Schattnerb Y, Berg E, Kivelsond SA (2017). Superconductivity and non-Fermi liquid behavior near a nematic quantum critical point. Proc. Natl. Acad. Sci. U.S.A..

[38] Licciardello S, Buhot J, Hu J, Ayres J, Kasahara S, Matasuda Y, Shibauchi T, Hussey NE (2019). Electrical resistivity across a nematic quantum critical point. Nature.

[39] Bristow M, Reiss P, Haghighirad AA, Zajicek Z, Singh SJ, Wolf T, Graf D, Knafo W, McCollam A, Coldea AI (2020). Anomalous high-magnetic field electronic state of the nematic superconductors FeSe$$_{1-x}$$S$$_x$$. Phys. Rev. Research.

[40] Huang WK, Hosoi S, Culo M, Kasahara S, Sato Y, Matsuura K, Mizukami Y, Berben M, Hussey NE, Kontani H, Shibauchi T, Matsuda Y (2020). Non-Fermi liquid transport in the vicinity of the nematic quantum critical point of superconducting FeSe$$_{1-x}$$S$$_x$$. Phys. Rev. Research.

[41] M. C̆ulo, M. Berben, Y.-T. Hsu, J. Ayres, R. D. H. Hinlopen, S. Kasahara, Y. Matsida, T. Shibauchi, N. E. Hussey, Putative Hall response of the strange metal component in FeSe$$_{1-x}$$S$$_x$$, Phys. Rev. Res. **3**, 023069 (2021)

[42] Rana K, Furukawa Y (2022). Relationship between nematicity, antiferromagnetic fluxtuations, and superconductivity in FeSe$$_{1-x}$$S$$_x$$ revealed by NMR. Front. Phys..

[43] Zhang W, Wu S, Kasahara S, Shibauchi T, Matsuda Y, Blumberg G (2021). Quadrupolar charge dynamics in the nonmagnetic FeSe$$_{1-x}$$S$$_x$$ superconductors. PNAS.

[44] Lazarević N, Baum A, Milosavljević A, Peis L, Stumberger R, Bekaert J, Šolajić A, Pešić J, Wang A, Šćepanović M, Abeykoon AMM, Milošević MV, Petrovic C, Popović ZV, Hackl R (2022). Evolution of lattice, spin, and charge properties across the phase diagram of FeSe$$_{1-x}$$S$$_x$$. Phys. Rev. B.

[45] Coldea AI, Blake SF, Kasahara S, Haghighirad AA, Watson MD, Knafo W, Choi ES, McCollam A, Reiss P, Yamashita T, Bruma M, Speller SC, Matsuda Y, Wolf T, Shibauchi T, Schofield AJ (2019). Evolution of the low-temperature Fermi surface of superconducting FeSe$$_{1-x}$$S$$_x$$ across a nematic phase transition, npj Quant. Mater..

[46] Sefat AS, McGuire MA, Sales BC, Jin R, Howe JY, Mandrus D (2008). Electronic correlations in the superconductor LaFeAsO$$_{0.89}$$F$$_{0.11}$$ with low carrier density. Phys. Rev. B.

[47] Kang N, Auban-Senzier P, Pasquier CR, Ren ZA, Yang J, Chen GC, Zhao ZX (2009). Pressure dependence of the thermoelectric power of the iron-based high-$$T_c$$ superconductor SmFeAsO$$_{0.85}$$. New J. Phys..

[48] E. D. Mun, S. L. Bud$$^\prime$$ko, Ni Ni, A. N. Thaler, P. C. Canfield, Thermoelectric power and Hall coefficient measurements on Ba(Fe$$_{1-x}$$T$$_x$$)$$_2$$As$$_2$$ (T = Co and Cu), Phys. Rev. B **80**, 054517 (2009)

[49] Pourret A, Malone L, Antunes AB, Yadav CS, Paulose PL, Fauque B, Behnia K (2011). Strong correlation and low carrier density in Fe$$_{1+y}$$Te$$_{0.6}$$Se$$_{0.4}$$ as seen from its thermoelectric response. Phys. Rev. B.

[50] Matusiak M, Plackowski T, Bukowski Z, Zhigadlo ND, Karpinski J (2009). Evidence of spin-density-wave order in RFeAsO$$_{1-x}$$F$$_x$$ from measurements of thermoelectric power. Phys. Rev. B.

[51] Butch NP, Saha SR, Zhang XH, Kirshenbaum K, Greene RL, Paglione J (2010). Effective carrier type and field dependence of the reduced-$$T_c$$ superconducting state in SrFe$$_{2-x}$$Ni$$_x$$As$$_2$$. Phys. Rev. B.

[52] Wang K, Lei H, Petrovic C (2011). Thermoelectric studies of K$$_x$$Fe$$_{2-y}$$Se$$_2$$ indicating a weakly correlated superconductor. Phys. Rev. B.

[53] Wang K, Lei H, Petrovic C (2011). Evolution of correlation strength in K$$_x$$Fe$$_{2-y}$$Se$$_2$$ superconductor doped with S. Phys. Rev. B.

[54] Collignon C, Ataei A, Gourgout A, Badoux S, Lizaire M, Legros A, Licciardello S, Wiedmann S, Yan JQ, Zhou JS, Ma Q, Gaulin BD, Doiron-Leyraud N, Tillefer L (2021). Thermopower across the phase diagram of the cuprate La$$_{1.6-x}$$Nd$$_{0.4}$$Sr$$_x$$CuO$$_4$$: Signatures of the pseudogap and charge density wave phases. Phys. Rev. B.

[55] Kim D, Shin E-C, Lee Y, Lee YH, Zhao M, Kim Y-H, Yang H (2022). Atomic-scale thermopower in charge density wave states. Nat. Commun..

[56] Mandal PR, Sarkar T, Greene RL (2019). Anomalous quantum criticality in the electron-doped cuprates. Proc. Natl. Acad. Sci. U.S.A..

[57] Badoux S, Afshar SAA, Michon B, Ouellet A, Fortier S, LeBoeuf D, Croft TP, Lester C, Hayden SM, Takagi H, Yamada K, Graf D, Doiron-Leyraud N, Taillefer L (2016). Critical doping for the onset of Fermi-surface reconstruction by charge-density-wave order in the cuprate superconductor La$$_{2-x}$$Sr$$_x$$CuO$$_4$$. Phys. Rev. X.

[58] Cyr-Choinière O, Badoux S, Grissonnanche G, Michon B, Afshar SAA, Fortier S, LeBoeuf D, Graf D, Day J, Bonn DA, Hardy WN, Liang R, Doiron-Leyraud N, Taillefer L (2017). Anisotropy of the Seebeck coefficient in the cuprate superconductor YBa$$_2$$Cu$$_3$$O$$_y$$: Fermi-surface reconstruction by bidirectional charge order. Phys. Rev. X.

[59] Skornyakov SL, Leonov AI (2019). Correlated electronic structure, orbital-dependent correlations and Lifshitz transition in tetragonal FeS. Phys. Rev. B.

[60] Wang A, Wu L, Ivanovski VN, Warren JB, Tian J, Zhu Y, Petrovic C (2016). Critical current density and vortex pinning in tetragonal FeS$$_{1-x}$$Se$$_x$$ (x = 0, 0.06). Phys. Rev. B.

[61] Wang A, Petrovic C (2017). Vortex pinning and irreversibility fields in FeS$$_{1-x}$$Se$$_x$$ (x = 0, 0.06). Appl. Phys. Lett..

[62] Liu Y, Wang A, Ivanovski VN, Du Q, Koteski V, Petrovic C (2022). Thermoelectricity and electronic correlation enhancement in FeS by light Se doping. Phys. Rev. B.

[63] Wang A, Milosavljevic A, Abeykoon AMM, Ivanovski V, Du Q, Baum A, Stavitski E, Liu Y, Lazarevic N, Attenkofer K, Hackl R, Popovic Z, Petrovic C (2022). Suppression of superconductivity and nematic order in Fe$$_{1-y}$$Se$$_{1-x}$$S$$_x$$ ($$0 \le x \le 1, y \le 0.1$$) crystals by anion height disorder. Inorg. Chem..

[64] Wang A, Wu L, Du Q, Naamneh M, Brito WH, Abeykoon AMM, Pudelko WR, Jandke J, Liu Y, Plumb NC, Kotliar G, Dobrosavljevic V, Radovic M, Zhu Y, Petrovic C (2022). Mooij law violation from nanoscale disorder. Nano Lett..

[65] Wang A, Petrovic C (2022). Electron correlations in the $$H_{c2}$$ of Fe$$_y$$Se$$_{1-x}$$S$$_x$$ ($$0.10 \le x \le 0.24, y \ge 0.9$$). Supercond. Sci. Technol..

[66] Watson MD, Kim TK, Haghighirad AA, Blake SF, Davies NR, Hoesch M, Wolf T, Coldea AI (2015). Suppression of orbital ordering by chemical pressure in FeSe$$_{1-x}$$S$$_x$$. Phys. Rev. B.

[67] Böhmer AE, Taufour V, Straszheim WE, Wolf T, Canfield PC (2016). Variation of transition temperatures and residual resistivity ratio in vapor-grown FeSe. Phys. Rev. B.

[68] Kasahara S, Watashige T, Hanaguri T, Kohsaka Y, Yamashita T, Shimoyama Y, Mizukami Y, Endo R, Ikeda H, Aoyama K, Terashima T, Uji S, Wolf T, Löhneysen HV, Shibauchi T, Matsuda Y (2014). Field-induced superconducting phase of FeSe in the BCS-BES cross-over. PNAS.

[69] Sun Y, Kittaka S, Nakamura S, Sakakibara T, Irie K, Namoto T, Machida K, Chen J, Tamegai T (2017). Gap structure of FeSe determined by angle-resolved specific heat measurements in applied rotating magnetic field. Phys. Rev. B.

[70] Lai X, Zhang H, Wang Y, Wang X, Zhang X, Lin J, Huang F (2015). Observation of superconductivity in tetragonal FeS. J. Am. Chem. Soc..

[71] Borg CKH, Zhou X, Eckberg C, Campbell DJ, Saha SR, Paglione J, Rodriguez EE (2016). Strong anisotropy in nearly ideal tetrahedral superconducting FeS single crystals. Phys. Rev. B.

[72] Lin H, Li Y, Deng Q, Xing J, Liu J, Zhu X, Yang H, Wen HH (2016). Multiband superconductivity and large anisotropy in FeS crystals. Phys. Rev. B.

[73] Terashima T, Kikugawa N, Lin H, Zhu X, Wen H-H, Nomoto T, Suzuki K, Ikeda H, Uji S (2016). Upper critical field and quantum oscillations in tetragonal superconducting FeS. Phys. Rev. B.

[74] Terashima T, Kikugawa N, Kiswandhi A, Choi E-S, Brooks J, Kasahara S, Watashige T, Ikeda H, Shibauchi T, Matsuda Y, Wolf T, Bohmer AE, Hardy F, Meingast C, Lohneysen HV, Suzuki M, Arita R, Uji S (2014). Anomalous Fermi surface in FeSe seen by Shubnikov-de-Haas oscillation measurements. Phys. Rev. B.

[75] Nabeshima F, Ishikawa T, Oyanagi K, Kawai M, Maeda A (2018). Growth of superconducting epitaxial films of sulfur substituted FeSe via pulsed laser deposition. J. Phys. Soc. Jpn..

[76] Das T, Panagopoulos C (2016). Two types of superconducting domes in unconventional superconductors. New J. Phys..

[77] Li L, Deng X, Wang Z, Liu Y, Abeykoon M, Dooryhee E, Tomic A, Huang Y, Warren JB, Bozin ES, Billinge SJL, Sun Y, Zhu Y, Kotliar G, Petrovic C (2017). Superconducting order from disorder in 2H-Ta$$_{2-x}$$Se$$_x$$,. NPJ Quant. Mater..

[78] Zhu CC, Yang XF, Xia W, Yin QW, Wang LS, Zhao CC, Dai DZ, Tu CP, Song BQ, Tao ZC, Tu ZJ, Gong CS, Lei HC, Guo YF, Li SY (2022). Double-dome superconductivity under pressure in the V-based kagome metals AV$$_3$$Sb$$_5$$ (A = Rb and K). Phys. Rev. B.

[79] Chen KY, Wang NN, Yin QW, Gu YH, Jiang K, Tu ZJ, Gong CS, Uwatoko Y, Sun JP, Lei HC, Hu JP, Cheng JG (2021). Double supercondcuting dome and triple enhancement of $$T_c$$ in the Kagome superconductor CsV$$_3$$Sb$$_5$$ under high pressure. Phys. Rev. Lett..

[80] Watson MD, Yamashita T, Kasahara S, Knafo W, Nardone M, Béard J, Hardy F, McCollam A, Narayanan A, Blake SF, Wolf T, Haghighirad AA, Meingast C, Schofield AJ, Löhneysen HV, Matsuda Y, Coldea AI, Shibauchi T (2015). Dichotomy between the hole and electron behavior in multiband superconductor FeSe probed by ultrahigh magnetic fields. Phys. Rev. Lett..

[81] Sun Y, Pyon S, Tamegai T (2016). Electron carriers with possible Dirac-cone-like dispersion in FeSe$$_{1-x}$$S$$_x$$ ($$x$$ = 0 and 0.14) single crystals triggered by structural transition. Phys. Rev. B.

[82] Huynh KK, Tanabe Y, Urata T, Oguro H, Heguri S, Watanabe K, Tanigaki K (2014). Electric transport of a single-crystal iron chalcogenide FeSe superconductor: evidence of symmetry-breakdown nematicity and additional ultrafast Dirac cone-like carriers. Phys. Rev. B.

[83] Song YJ, Hong JB, Min BH, Kwon YS, Lee KJ, Jung MH, Rhyee JS (2011). Superconducting properties of a stoichiometric FeSe compound and two anomalous features in the normal state. J. Korean Phys. Soc..

[84] Caglieris F, Ricci F, Lamura G, Martinelli A, Palenzona A, Pallecchi I, Sala A, Profeta G, Putti M (2012). Theoretical and experimental investigation of magnetotransport in iron chalcogenides. Sci. Technol. Adv. Mater..

[85] Onar K, Yakinci ME (2015). Solid state synthesis and characterization of bulk $$\beta$$-FeSe superconductors. J. Alloy. Compd..

[86] Yi M, Pfau H, Zhang Y, He Y, Wu H, Chen T, Ye ZR, Hashimoto M, Yu R, Si Q, Lee D-H, Dai P, Shen Z-X, Lu DH, Birgeneau RJ (2019). Nematic energy scale and the missing electron pocket in FeSe. Phys. Rev. X.

[87] Mandal S, Cohen RE, Haule K (2014). Strong pressure-dependent electron-phonon coupling in FeSe. Phys. Rev. B.

[88] Q. Du, L. Wu, H. Cao, C.-J. Kang, C. Nelson, G.L. Pascut, T. Besara, T. Siegrist, K. Haule, G. Kotliar, I. Zaliznyak, Y. Zhu, C. Petrovic, Vacancy defect control of colossal thermopower in FeSb2. npj. Quantum Mater. **6**, 13 (2021)

[89] Hafiez MA, Pu YJ, Brisbois J, Peng R, Feng DL, Chareev DA, Silhanek AV, Krellner C, Vasiliev AN, Chen XJ (2016). Impurity scattering effects on the superconducting properties and the tetragonal-to-orthorhombic phase transition in FeSe. Phys. Rev. B.

[90] Barnard RD (1972). Thermoelectricity in metals and alloys.

[91] Cohn JL, Wolf SA, Selvamanickam V, Salama K (1991). Thermoelectric power of YBa$$_2$$Cu$$_3$$O$$_{7-\delta }$$: Phonon drag and multiband conduction. Phys. Rev. Lett..

[92] Behnia K, Jaccard D, Flouquet J (2004). On the thermoelectricity of correlated electrons in the zero-temperature limit. J. Phys. Condens. Matter..

[93] Miyake K, Kohno H (2005). Theory of quasi-universal ratio of seebeck coefficient to specific heat in zero-temperature limit in correlated metals. J. Phys. Soc. Jpn..

[94] Jiang S, Jeevan HS, Dong J, Gegenwart P (2013). Thermopower as a sensitive probe of electronic nematicity in iron pnictides. Phys. Rev. Lett..

[95] McMillan WL (1968). Transition temperature of strong-coupled superconductors. Phys. Rev..

[96] Xing J, Lin H, Li Y, Li S, Zhu X, Yang H, Wen HH (2016). Nodal superconducting gap in tetragonal FeS. Phys. Rev. B.

[97] Hrischfeld P, Korshunov M, Mazin I (2011). Gap symmetry and structure of Fe-based superconductors. Rep. Prog. Phys..

